# Plain Water and Sugar-Sweetened Beverage Consumption in Relation to Energy and Nutrient Intake at Full-Service Restaurants

**DOI:** 10.3390/nu8050263

**Published:** 2016-05-04

**Authors:** Ruopeng An

**Affiliations:** Department of Kinesiology and Community Health, College of Applied Health Sciences, University of Illinois at Urbana-Champaign, Champaign, IL 61820, USA; ran5@illinois.edu; Tel.: +1-217-244-0966

**Keywords:** plain water, sugar-sweetened beverage, diet quality, 24-h dietary recall, full-service restaurant, energy intake, added sugar, saturated fat, sodium

## Abstract

Background: Drinking plain water, such as tap or bottled water, provides hydration and satiety without adding calories. We examined plain water and sugar-sweetened beverage (SSB) consumption in relation to energy and nutrient intake at full-service restaurants. Methods: Data came from the 2005–2012 National Health and Nutrition Examination Survey, comprising a nationally-representative sample of 2900 adults who reported full-service restaurant consumption in 24-h dietary recalls. Linear regressions were performed to examine the differences in daily energy and nutrient intake at full-service restaurants by plain water and SSB consumption status, adjusting for individual characteristics and sampling design. Results: Over 18% of U.S. adults had full-service restaurant consumption on any given day. Among full-service restaurant consumers, 16.7% consumed SSBs, 2.6% consumed plain water but no SSBs, and the remaining 80.7% consumed neither beverage at the restaurant. Compared to onsite SSB consumption, plain water but no SSB consumption was associated with reduced daily total energy intake at full-service restaurants by 443.4 kcal, added sugar intake by 58.2 g, saturated fat intake by 4.4 g, and sodium intake by 616.8 mg, respectively. Conclusion: Replacing SSBs with plain water consumption could be an effective strategy to balance energy/nutrient intake and prevent overconsumption at full-service restaurant setting.

## 1. Introduction

Eating out has become an essential part of the American diet [1,2]. A long line of existing studies have documented fast-food restaurant consumption in relation to increased energy intake and elevated risk of obesity in children and adults [3,4,5,6,7,8,9,10,11,12,13]. Accumulating evidence suggests that full-service restaurant consumption shares similar, if not more concerning, nutrition implications as fast-food restaurant consumption [14,15]. Given that approximately one fifth of U.S. adults eat in a full-service restaurant on any given day [14], reducing energy intake and improving diet quality in full-service restaurant settings may profoundly impact Americans’ nutritional and health status.

Adequate hydration is essential to body function [16]. Drinking plain water, such as tap or bottled water, delivers adequate hydration without adding calories [17]. Plain water intake has been linked to reduced energy consumption and improved body weight management [18,19,20,21]. Potential mechanisms include, but may not be limited to, plain water intake in substitution for caloric beverage consumption [22], and satiety from plain water consumption in coping with feelings of hunger and desire to eat [23]. The 2015–2020 Dietary Guidelines for Americans recommended “choosing beverages with no added sugars, such as water, in place of sugar-sweetened beverages” as an effective strategy to reduce added sugar consumption [24]. In addition, beverages are often consumed together with other foods, which jointly impact daily total calorie intake and overall diet quality. Using individual fixed-effects model based on two non-consecutive NHANES 24-h dietary recall data, An (2016) documented that in comparison to the days when no sugar-sweetened beverages (SSBs) were consumed, participants tended to consume more discretionary foods—foods that are typically low in nutrient value but high in added sugar, sodium, saturated fats, and cholesterol on days when they consumed SSBs [25].

Plain water is often available and free-of-charge at a full-service restaurant. Drinking plain water in substitution for SSBs could contribute to the reduction of total energy intake and intake of certain nutrients that are of major public health concern, such as added sugar when dining at a full-service restaurant. To our knowledge, no study has examined the nutritional implications of plain water consumption at full-service restaurant setting. Using in-person 24-h dietary recall data from a nationally-representative repeated cross-sectional health survey, this study assessed plain water and SSB consumption in relation to energy and nutrient intake at full-service restaurants among U.S. adults.

## 2. Materials and Methods

### 2.1. Survey Setting

The National Health and Nutrition Examination Survey (NHANES) is a program of studies conducted by the National Center for Health Statistics (NCHS) to assess the health and nutritional status of children and adults. The program began in the early 1960s and periodically conducted separate surveys focusing on different population groups or health topics. Since 1999, the NHANES has been conducted continuously in two-year cycles and has a changing focus on a variety of health and nutrition measurements. A multistage probability sampling design is used to select participants representative of the civilian, non-institutionalized U.S. population. Certain population subgroups are oversampled to increase the reliability and precision of health status indicator estimates for these groups. Detailed information regarding the NHANES sampling design, questionnaires, clinical measures, and individual-level data, can be found on its web portal [26].

### 2.2. Dietary Recall

Starting from the NHANES 1999–2000 wave, all participants were asked to complete an in-person 24-h dietary recall (a subsequent telephone-based dietary recall was added since 2001–2002 wave and data became publicly available since 2003–2004 wave). In the dietary recall, each food/beverage item and corresponding quantity consumed by a participant from midnight to midnight on the day before the recall was recorded. The in-person dietary recall was conducted by trained dietary interviewers in the Mobile Examination Center with a standard set of measuring guides. These tools aimed to help the participant accurately report the volume and dimensions of the food/beverage items consumed. Following the dietary recall, the energy and nutrient contents of each reported food/beverage item were systematically coded with the U.S. Department of Agriculture’s Food and Nutrient Database for Dietary Studies (FNDDS).

Following An and McCaffrey [27] and Drewnowski *et al.* [28,29], this study used individual-level data from the NHANES 2005–2006, 2007–2008, 2009–2010, and 2011–2012 waves. Those waves were chosen because the collection of data on tap and bottled water consumption as a beverage only started in 2005 as part of the 24-h dietary recall, whereas in previous waves, such information was assessed via questionnaire after the 24-h dietary recall was completed.

### 2.3. Plain Water Consumption

Following An and McCaffrey (2016) [27] and Drewnowski *et al.* (2013a, b) [28,29], plain water consumption includes intake of plain tap water, water from a drinking fountain, water from a water cooler, bottled water, and spring water. In the NHANES 2005–2012 waves, the FNDDS codes 94000100 (“water, tap”) and 94100100 (“water, bottled, unsweetened”) were used to identify plain water consumption.

### 2.4. Sugar-Sweetened Beverage Consumption

SSBs include sodas, fruit drinks, energy drinks, sports drinks, and sweetened bottled waters, consistent with definitions reported by the Centers for Disease Control and Prevention (CDC) and the National Cancer Institute (NCI) [30]. In the NHANES 2011–2012 wave, SSBs consist of 48 reported beverage items. The number of reported items in the SSB category differed only slightly across survey waves.

### 2.5. Consumption of Other Beverage Types

In addition to plain water and SSBs, consumption of other beverage types including diet beverage, coffee, tea, alcohol, juice, and milk were summarized in descriptive statistics. Diet beverage includes calorie-free and low-calorie versions of sodas, fruit drinks, energy drinks, sports drinks, and carbonated water consistent with definitions reported by the CDC, NCI, and the Food and Drug Administration food labeling guidelines [31,32,33]. Coffee includes any form of regular or decaffeinated coffee product or coffee substitute (e.g., cereal grain beverage). Tea includes any form of regular or decaffeinated tea product. Alcohol includes beers and ales, cordials and liqueurs, cocktails, wines, and distilled liquors. The definitions on coffee, tea and alcohol are consistent with the USDA FNDDS food/beverage categorization [25]. Beverages in the juice and milk categories were identified based on the Food Patterns Equivalents Database (FPED), which were linked to the NHANES 24-h dietary recall data.

### 2.6. Added Sugar Consumption

Added sugar is sugar that is not naturally found in a food product but is added during the food production process. The USDA uses ingredient list and total sugar amounts provided to estimate the quantity of added sugar in a food product [34]. We used the FPED which contains the estimated added sugar amounts for each food/beverage consumed by the NHANES 24-h dietary recall participants.

### 2.7. Onsite Full-Service Restaurant Consumption

The NHANES dietary interviews asked about the source (e.g., restaurant, store, vending machine) of each food/beverage item consumed on a dietary recall day, and also whether the item was consumed at home or away from home. Following An [14] and Powell *et al.* [15], consumption of a food/beverage item qualified for an onsite full-service restaurant consumption if the item was obtained from a “restaurant with waiter/waitress” and consumed away from home.

In the dietary recall data, energy/nutrient derived from each consumed food/beverage item was recorded based on the quantity of food/beverage reported and the corresponding energy/nutrient contents. We calculated daily energy (kcal) and plain water (g), SSBs (g), added sugar (g), saturated fat (g), and sodium (mg) consumed onsite at a full-service restaurant among those NHANES participants who reported any onsite full-service restaurant consumption in the in-person 24-h dietary recall. We further classified full-service restaurant consumers into three mutually-exclusive categories based on their onsite SSB and plain water consumption status—SSB consumption (consumption of any positive grams of SSBs at a full-service restaurant), plain water but no SSB consumption (consumption of any positive grams of plain water but zero grams of SSBs at a full-service restaurant), and no plain water or SSB consumption (consumption of zero grams of plain water and SSBs at a full-service restaurant).

Among a total of 19,245 U.S. adults 18 years of age and above who participated in the in-person 24-h dietary recalls in the NHANES 2005–2012 waves, 934 who were pregnant, lactating, and/or on a special diet to lose weight at the time of interview were excluded. Of the remaining 18,311 participants, 2900 reported onsite full-service restaurant consumption on the dietary recall day. Among full-service restaurant consumers, 523 had SSB consumption, 85 had plain water but no SSB consumption, and the remaining 2292 had no plain water or SSB consumption at the restaurant.

In the analyses, we combined two mutually-exclusive categories, namely SSB but no plain water consumption (consumption of any positive grams of SSBs but zero grams of plain water at a full-service restaurant) and SSB and plain water consumption (consumption of any positive grams of SSBs and plain water at a full-service restaurant), into one category *i.e.*, SSB consumption, because the category of SSB and plain water consumption comprised an insufficient sample size of merely 11. In sensitivity analyses, we regressed each outcome variable (daily energy intake and intake of added sugar, saturated fat, and sodium at a full-service restaurant) on all four categories based on onsite SSB and plain water consumption status (plain water but no SSB consumption, no plain water or SSB consumption, and SSB and plain water consumption, with SSB but no plain water consumption in the reference group). The estimated coefficients were almost identical as those based on the three categories (plain water but no SSB consumption, and no plain water or SSB consumption, with SSB consumption in the reference group). None of the coefficients pertaining to the category of SSB and plain water consumption were statistically significant in the sensitivity analyses.

### 2.8. Individual Characteristics

The following individual characteristics were adjusted for in regression analyses: a dichotomous variable for sex (female, with male in the reference group); three categorical variables for age groups (18–34 years of age, 35–49 years of age, and 50–64 years of age, with 65 years of age and above in the reference group); three categorical variables for race/ethnicity (non-Hispanic black, non-Hispanic other race or multi-race, and Hispanic, with non-Hispanic white in the reference group); a dichotomous variable for education attainment (college education and above, with high school or lower education in the reference group); two categorical variables for marital status (divorced or separated or widowed, and never married, with married in the reference group); two categorical variables for household income level (130% ≤ income to poverty ratio [IPR] < 300%, and IPR ≥ 300%, with IPR < 130% in the reference group; IPR is the ratio of annual household income to poverty level specified in the Department of Health and Human Services’ poverty guidelines); a dichotomous variable for body weight status (obesity defined as body mass index [BMI] ≥ 30 kg/m^2^ based on the international classification of adult BMI values [35], with non-obesity in the reference group); a dichotomous variable for smoking status (ever or current smoker, with never smoking in the reference group); a dichotomous variable for self-rated health (good or excellent self-rated health, with poor or fair self-rated health in the reference group); five dichotomous variables for each of the chronic condition diagnoses *i.e.*, diabetes, arthritis, coronary heart disease, stroke, and cancer; a dichotomous variable for day of the week (weekend days including Friday, Saturday, and Sunday, with weekdays including Monday, Tuesday, Wednesday, and Thursday in the reference group) [36]; and three categorical variables for the NHANES waves (2007–2008, 2009–2010, and 2011–2012 waves, with 2005–2006 wave in the reference group).

### 2.9. Statistical Analyses

We summarized individual characteristics and daily energy/nutrient at full-service restaurants among the 2005–2012 NHANES adult full-service restaurant consumers by onsite SSB and plain water consumption status in descriptive statistics. Linear regressions were performed to estimate the differences in energy/nutrient intake at full-service restaurants by SSB and plain water consumption status, adjusting for individual characteristics. The four outcome variables were daily intake of energy (kcal), added sugar (g), saturated fat (g), and sodium (mg) at a full-service restaurant. Reductions of daily total energy intake and intake of added sugar, saturated fat, and sodium have been the key recommendations of the 2015–2020 Dietary Guidelines for Americans [24]. The key independent variables were two categorical variables for SSB and plain water consumption status at full service restaurant (plain water but no SSB consumption, and no plain water or SSB consumption, with SSB consumption in the reference group).

The dose-response relationship between SSB and plain water consumption and energy/nutrient intake at a full-service restaurant was assessed by regressing the outcome variables on the continuous variables for quantities (g) consumed of SSBs and plain water, adjusting for individual characteristics.

The NHANES 2005–2012 multi-wave sampling design was accounted for in both descriptive statistics and regression analyses. Specifically, we followed the NCHS instructions to construct sampling weights when combining survey waves [37]. We then applied the “svy” commands in Stata to specify sampling weights, sampling strata, and primary sampling units, as well as to conduct regression analyses. All statistical procedures were performed in Stata 14.1 SE version (StataCorp, College Station, TX, USA).

### 2.10. Human Subjects Protection

The NHANES was approved by the NCHS Research Ethics Review Board. This study used the NHANES de-identified public data and was deemed exempt from human subjects review by the University of Illinois at Urbana-Champaign Institutional Review Board.

## 3. Results

During 2005–2012, approximately 18.2% of U.S. adults had onsite full-service restaurant consumption on any given day. Among full-service restaurant consumers, 16.7% had SSB consumption, 2.6% had plain water but no SSB consumption, and the remaining 80.7% had no plain water or SSB consumption at the restaurant. Those who had SSB consumption on average consumed 612.0 g of SSB, and those who had plain water but no SSB consumption consumed 639.3 g of plain water at the restaurant. Among those who consumed no plain water or SSB at the restaurant, the prevalence of diet beverage, coffee, tea, alcohol, juice, and milk consumption (mutually-unexclusive) were 9.8%, 13.8%, 21.3%, 14.6%, 17.4%, and 24.1%, respectively.

Table 1 reports individual characteristics of adult full-service restaurant consumers by onsite SSB and plain water consumption status. Compared to women, men were more likely to consume SSBs (63.3%) but less likely to consume plain water (43.1%) in a full-service restaurant. SSB consumers consisted of a larger share of younger adults 18–34 years of age (40.8%) than plain water consumers (24.7%), whereas higher education was less prevalent among SSB consumers (58.6%) than among plain water consumers (67.7%). A smaller proportion of SSB consumers were at the lowest income level (IPR < 130%) but a larger proportion at the middle income level (130% ≤ IPR < 300%) compared to plain water consumers, whereas the prevalence of the highest income level (IPR ≥ 130%) remained similar between these two groups. Obesity rate and the prevalence of poor or fair self-rated health were slightly higher among SSB consumers compared to plain water consumers, and SSB consumers consisted of a larger proportion of former or current smokers.

Table 2 reports daily energy and nutrient intake at a full-service restaurant by onsite SSB and plain water consumption status. Among those three groups, SSB consumers had the highest daily intake of total energy, added sugar, saturated fat, and sodium from a full-service restaurant, whereas plain water consumers had the lowest, with those who consumed neither SSB nor water in between. Daily energy intake at a full-service restaurant totaled 1277.0 kcal among SSB consumers, 527.6 kcal, and 369.8 kcal higher than among plain water consumers and SSB/water nonconsumers, respectively. Daily added sugar intake at a full-service restaurant was 69.3 g among SSB consumers, 60.1 g and 54.5 g higher than among plain water consumers and SSB/water non-consumers, respectively. Daily saturated fat intake at a full-service restaurant was 15.0 g among SSB consumers, 5.7 g and 2.6 g higher than among plain water consumers and SSB/water non-consumers, respectively. Daily sodium intake at a full-service restaurant was 2331.0 mg among SSB consumers, 755.2 mg and 453.1 mg higher than among plain water consumers and SSB/water non-consumers, respectively.

Table 3 reports the adjusted differences in daily energy and nutrient intake at a full-service restaurant by onsite SSB and plain water consumption status based on regression estimates. After adjusting for individual characteristics, daily energy intake at a full-service restaurant among SSB consumers was 443.4 (95% confidence interval CI = 297.3, 589.6) kcal and 321.3 (95% CI = 224.0, 418.7) kcal higher than among plain water consumers and SSB/water non-consumers, respectively. Adjusted daily added sugar intake at a full-service restaurant among SSB consumers was 58.2 (95% CI = 52.2, 64.2) g and 53.3 (95% CI = 48.3, 58.4) g higher than among plain water consumers and SSB/water non-consumers, respectively. Adjusted daily saturated fat intake at a full-service restaurant among SSB consumers was 4.4 (95% CI = 2.0, 6.8) g and 2.2 (95% CI = 0.6, 3.8) g higher than among plain water consumers and SSB/water non-consumers, respectively. Adjusted daily sodium intake at a full-service restaurant among SSB consumers was 616.8 (95% CI = 286.8, 946.8) mg and 380.5 (95% CI = 160.8, 600.2) mg higher than among plain water consumers and SSB/water non-consumers, respectively.

Women consumed less daily total energy, added sugar, saturated fat, and sodium at a full-service restaurant than men. Compared to those 18–34 years of age, those 35–49 years of age consumed more sodium, whereas those 65 years of age and above consumed less total energy and saturated fat at a full-service restaurant. Compared to non-Hispanic whites, non-Hispanic blacks consumed less total energy, saturated fat, and sodium, Hispanics consumed less total energy, added sugar, and sodium, and non-Hispanic other race/multi-race consumed less added sugar and saturated fat at a full-service restaurant. Those with good or excellent self-rated health consumed less saturated fat at a full-service restaurant than those with poor or fair self-rated health. Those with coronary artery disease consumed less total energy and those with cancer consumed less sodium at a full-service restaurant than those without such chronic conditions. Full-service restaurant consumption during weekend days was associated with higher total energy and added sugar intake than consumption during weekdays. Compared to those with high school or lower education, those with college or higher education consumed less added sugar at a full-service restaurant. Daily total energy, added sugar, saturated fat, and sodium intake at a full-service restaurant were not found to be associated with marital status, income level, obesity, or smoking. No temporal trend in daily total energy, sugar, saturated fat, and sodium intake at a full-service restaurant was identified as none of the coefficients with respect to survey waves were statistically significant at *p* < 0.05.

A dose-response relationship between onsite SSB consumption and energy/nutrient intake at a full-service restaurant was identified, whereas such relationship pertaining to onsite plain water consumption was statistically significant for total energy, added sugar, and saturated fat intake but not for sodium intake. An increase in onsite SSB consumption by 100 g was associated with an increase in daily intake of total energy at a full-service restaurant by 65.1 (95% CI = 50.6, 79.5) kcal, added sugar by 9.3 (95% CI = 8.7, 9.8) g, saturated fat by 0.5 (95% CI = 0.2, 0.8) g, and sodium by 78.9 (95% CI = 44.0, 113.7) mg. An increase in onsite plain water consumption by 100 g was associated with a reduction in daily intake of total energy at a full-service restaurant by 17.8 (95% CI = 0.6, 35.0) kcal, added sugar by 0.5 (95% CI = 0.1, 0.8) g, saturated fat by 0.3 (95% CI = 0.0, 0.6) g, and sodium by 32.0 (95% CI = −6.1, 70.2) mg.

## 4. Discussion

This study examined plain water and SSB consumption in relation to energy and nutrient intake at full-service restaurants among U.S. adults, using 24-h dietary recall data from a nationally-representative health survey. Over 18% of U.S. adults had onsite full-service restaurant consumption on any given day during 2005–2012. Among full-service restaurant consumers, approximately 16.7% consumed SSBs, 2.6% consumed plain water but no SSBs, and the remaining 80.7% consumed neither beverage at the restaurant. Adjusting for individual characteristics and accounting for sampling design, those consuming SSBs onsite had the highest daily intake of total energy, added sugar, saturated fat, and sodium at a full-service restaurant, whereas those consuming plain water but no SSBs had the lowest, with those consuming neither beverage was in between. Compared to onsite SSB consumption, plain water but no SSB consumption was associated with a reduction in daily total energy intake at a full-service restaurant by 443.4 kcal, added sugar intake by 58.2 g, saturated fat intake by 4.4 g, and sodium intake by 616.8 mg, respectively.

Replacing SSBs with plain water consumption has shown to be associated with reduced daily intake of total energy, added sugar, saturated fat, and sodium in some intervention and epidemiological studies [38,39,40]. Dining out at a full-service restaurant has been linked to increased energy intake and reduced diet quality [14,15,41]. Findings from this study confirmed the beneficial nutritional implications of plain water consumption at the full-restaurant setting. Drinking plain water in substitution for SSBs could help cut total calories as well as intake of certain nutrients that are of major public health concern, such as added sugar, saturated fat, and sodium when dining at a full-service restaurant.

Despite the nutritional desirability of replacing SSBs with plain water consumption, only a tiny proportion of full-service consumers chose to drink plain water, whereas the prevalence of onsite SSB consumption remained over five-fold larger. The lack of popularity in plain water consumption contrasts the fact that plain water is often easily accessible and made free-of-charge at a full-service restaurants as a “default” service to diners. Compared to eating at a fast-food restaurant, where plain water can be hard to find and a combo meal that includes SSBs is served as the “norm”, it could be more convenient and advantageous for full-service restaurant consumers to balance their dietary intake and prevent overconsumption through onsite plain water consumption.

A dose-response relationship between onsite SSB and plain water consumption and energy/nutrient intake at a full-service restaurant was identified (although the associations between plain water consumption and sodium intake were statistically nonsignificant). Higher levels of onsite SSB consumption were associated with increased daily intake of total energy, added sugar, saturated fat, and sodium at a full-service restaurant; whereas higher levels of plain water consumption were associated with reduced intake of total energy and saturated fat. Plain water consumption delivers satiety and reduces the feelings of hunger and desire to eat but adds no calories to one’s diet [17]. This study finding indicates that increasing onsite plain water consumption while refraining from SSB consumption could help achieve additional reduction in energy intake at full-service restaurant setting.

We restricted our classification of beverage consumption status at a full-service restaurant to the “dichotomy” of plain water and SSBs. This simplification allows us to examine the key contrasts of interest, which are based upon the accumulating evidence on the health benefits of plain water consumption and the detrimental impacts of SSB consumption on diet quality and obesity [42,43]. Arguably, other types of beverages (e.g., diet drinks, coffee, tea, alcohol, juice, and milk) also play important roles in determining energy and nutrient intake at full-service restaurants. For example, the prevalence of tea consumption is substantial, and if a large proportion of tea is calorically sweetened, it would constitute another large source of added sugar. However, a comprehensive examination of all beverage types is beyond the scope of this study.

A few limitations of this study should be noted. Dietary intake in the NHANES was self-reported and subject to measurement error and social desirability bias [44]. Prevalence of plain water consumption at full-service restaurants was low and this was possibly due to under-reporting of water intake. This study adopted a cross-sectional design. Although we attempted to reduce the influence of potential confounders by including a large set of covariates, it is possible that some unobserved differences in individual characteristics such as taste preferences and/or eating habits that are correlated with both outcomes and water/SSB consumption status at restaurants. A cross-sectional study design would not allow us to completely eliminate the possibility of confounding issue and, thus, the study findings warrant confirmation through controlled interventions. Despite use of multiple waves of data from a large nationally representative survey, only a tiny fraction (2.6%; *N* = 85) of the study sample drank plain water at a full-service restaurant, which compromised estimation precision and precluded further sample stratification and subgroup analyses by individual demographics and/or socioeconomic status. A dose-response relationship between onsite plain water consumption and sodium intake at a full-service restaurant was unidentified, possibly due to the very small sample size of plain water consumers and consequent lack of variations in quantities consumed. The NHANES is a probability sample of the U.S. non-institutionalized population, and patients in penal/mental facilities, institutionalized older adults, and/or military personnel on active duty are not represented.

## 5. Conclusions

Using 24-h dietary recall data from the 2005–2012 NHANES, this study assessed the relationship between plain water and SSB consumption and energy/nutrient intake at full-service restaurants in U.S. adults. Compared to onsite SSB consumption, plain water, but no SSB consumption, was associated with reduced daily intake of total calories, added sugar, saturate fat, and sodium at full-service restaurants. In comparison to home-prepared meals, dining out is prone to overeating and poorer dietary quality. Given that plain water is mostly available and free-of-charge at full-service restaurants, restricting one’s beverage consumption to only plain water when dining out could be a cost-free and easily-adaptable strategy to balance energy and nutrient intake and prevent overconsumption.

## Figures and Tables

**Table 1:** Individual characteristics of 2005–2012 NHANES adult full-service restaurant consumers by plain water and sugar-sweetened beverage (SSB) consumption status.

Individual Characteristics (%)	SSB Consumption (95% CI)	Plain Water but No SSB Consumption (95% CI)	No Plain Water or SSB Consumption (95% CI)
Sample size	523	85	2292
**Sex**			
Male	63.3 (58.2, 68.4)	43.1 (30.2, 56.0)	50.6 (48.5, 52.6)
Female	36.7 (31.6, 41.8)	56.9 (44.0, 69.8)	49.4 (47.4, 51.5)
**Age group**			
18–34 years of age	40.8 (35.5, 46.1)	24.7 (12.2, 37.1)	25.3 (22.7, 27.9)
35–49 years of age	30.5 (24.7, 36.4)	25.1 (12.9, 37.3)	30.7 (28.4, 33.1)
50–64 years of age	22.7 (17.5, 27.9)	38.6 (25.1, 52.1)	28.1 (25.3, 30.8)
65 years of age and above	6.0 (3.8, 8.1)	11.6 (2.3, 20.9)	15.9 (14.0, 17.8)
**Race/ethnicity**			
White, non-Hispanic	62.5 (56.2, 68.9)	67.2 (51.7, 82.7)	80.0 (77.2, 82.8)
Black, non-Hispanic	9.5 (6.9, 12.2)	5.2 (1.7, 8.7)	5.6 (4.4, 6.8)
Other race/multi-race, non-Hispanic	6.6 (4.1, 9.0)	8.2 (0.0, 16.9)	5.3 (4.2, 6.4)
Hispanic	21.4 (16.4, 26.4)	19.5 (8.9, 30.0)	9.1 (7.0, 11.2)
**Education**			
High school and below	41.4 (34.3, 48.5)	32.3 (21.8, 42.9)	31.0 (27.9, 34.1)
College education and above	58.6 (51.5, 65.7)	67.7 (57.1, 78.2)	69.0 (65.9, 72.1)
**Marital status**			
Married	69.6 (64.3, 74.8)	57.9 (46.2, 69.6)	68.0 (65.4, 70.7)
Divorced, separated, or widowed	10.2 (7.3, 13.0)	21.1 (10.8, 31.5)	15.8 (14.0, 17.7)
Never married	20.3 (15.8, 24.8)	21.0 (10.1, 31.8)	16.2 (13.8, 18.6)
**Income to poverty ratio (IPR)**			
IPR < 130%	16.6 (11.9, 21.4)	25.90 (11.7, 40.0)	10.5 (8.8, 12.3)
130% ≤ IPR < 300%	27.0 (22.0, 32.1)	17.0 (7.0, 26.9)	24.2 (21.6, 26.9)
IPR ≥ 300%	56.4 (50.2, 62.5)	57.2 (43.3, 71.1)	65.2 (62.3, 68.1)
**Obesity**			
Non-obese (BMI < 30)	66.5 (61.5, 71.6)	68.5 (57.4, 79.6)	65.7 (62.8, 68.6)
Obese (BMI ≥ 30)	33.5 (28.4, 38.6)	31.5 (20.4, 42.6)	34.3 (31.4, 37.2)
**Smoking**			
Non-smoker	61.2 (55.1, 67.4)	68.6 (53.8, 83.4)	57.4 (54.6, 60.2)
Former or current smoker	38.8 (32.6, 44.9)	31.4 (16.6, 46.2)	42.6 (39.9, 45.4)
**Self-rated health**			
Good or excellent health	86.4 (82.9, 89.8)	91.8 (86.8, 96.9)	89.0 (87.3, 90.6)
Fair or poor health	13.7 (10.2, 17.1)	8.2 (3.1, 13.2)	11.1 (9.4, 12.7)
**Chronic condition**			
Diabetes	6.9 (3.4, 10.3)	3.4 (0.6, 6.2)	8.4 (7.0, 9.7)
Arthritis	12.3 (9.0, 15.6)	17.1 (6.8, 27.3)	22.1 (20.0, 24.3)
Coronary artery disease	2.5 (0.5, 4.4)	0.4 (0.0, 1.3)	3.3 (2.4, 4.2)
Stroke	2.2 (0.4, 4.1)	2.7 (0.0, 5.9)	1.5 (0.9, 2.1)
Cancer	4.4 (2.1, 6.7)	12.2 (4.3, 20.0)	9.6 (7.9, 11.3)
**Day of the week**			
Weekday	27.3 (22.4, 32.2)	34.6 (13.9, 55.4)	33.6 (30.9, 36.2)
Weekend	72.7 (67.8, 77.6)	65.4 (44.7, 86.1)	66.4 (63.8, 69.1)
**Survey wave**			
2005–2006	28.0 (21.8, 34.2)	21.5 (7.4, 35.5)	25.2 (21.1, 29.3)
2007–2008	25.0 (17.1, 32.9)	22.4 (9.9, 35.0)	25.9 (22.8, 29.1)
2009–2010	20.2 (13.6, 26.9)	23.6 (10.2, 37.0)	23.2 (20.2, 26.2)
2011–2012	26.8 (18.1, 35.5)	32.6 (10.2, 54.9)	25.7 (22.2, 29.1)

Notes: The NHANES multi-wave sampling design was accounted for in estimating the percentages.

**Table 2:** Daily energy and nutrient intake at full-service restaurant by plain water and sugar-sweetened beverage (SSB) consumption status, 2005–2012 NHANES.

Intake	SSB Consumption (95% CI)	Plain Water but No SSB Consumption (95% CI)	No Plain Water or SSB Consumption (95% CI)
Sample size	523	85	2292
Total energy (kcal)	1277.0 (1184.3, 1369.6)	749.4 (613.5, 885.3)	907.2 (877.2, 937.1)
Added sugar (g)	69.3 (64.3, 74.2)	9.2 (6.2, 12.2)	14.8 (13.6, 16.0)
Saturated fat (g)	15.0 (13.4, 16.5)	9.3 (7.4, 11.2)	12.4 (12.0, 12.9)
Sodium (mg)	2331.0 (2122.0, 2540.1)	1575.8 (1243.4, 1908.2)	1877.9 (1819.8, 1936.1)

Notes: The NHANES multi-wave sampling design was accounted for in estimating the percentages.

**Table 3:** Adjusted differences in daily energy and nutrient intake at full-service restaurants by plain water and sugar-sweetened beverage (SSB) consumption status, 2005–2012 NHANES.

Independent Variable	Total Energy (kcal) (95% CI)	Added Sugar (g) (95% CI)	Saturated Fat (g) (95% CI)	Sodium (mg) (95% CI)
Sample size	2900	2900	2900	2900
**Plain water and SSB intake status**				
SSB consumption	Reference	Reference	Reference	Reference
Plain water but no SSB consumption	−443.4 *** (−589.6, −297.3)	−58.2 *** (−64.2, −52.2)	−4.4 ** (−6.8, −2.0)	−616.8 *** (−946.8, −286.8)
No plain water or SSB consumption	−321.3 *** (−418.7, −224.0)	−53.3 *** (−58.4, −48.3)	−2.2 ** (−3.8, −0.6)	−380.5 ** (−600.2, −160.8)
**Sex**				
Male	Reference	Reference	Reference	Reference
Female	−300.8 *** (−351.0, −250.6)	−6.0 *** (−8.3, −3.7)	−3.9 *** (−4.8, −3.0)	−581.3 *** (−691.0, −471.6)
**Age group**				
18–34 years of age	Reference	Reference	Reference	Reference
35–49 years of age	46.2 (−34.3, 126.7)	1.4 (−1.8, 4.6)	1.0 (−0.4, 2.3)	254.8 * (55.7, 453.8)
50–64 years of age	−41.2 (−120.8, 38.5)	2.0 (−2.2, 6.1)	−1.0 (−2.4, 0.3)	−6.3 (−171.8, 159.2)
65 years of age and above	−131.8 * (−231.3, −32.2)	−2.6 (−7.0, 1.8)	−2.2 ** (−3.9, −0.6)	−162.5 (−357.8, 32.8)
**Race/ethnicity**				
White, non-Hispanic	Reference	Reference	Reference	Reference
Black, non-Hispanic	−118.1 ** (−187.2, −49.0)	2.2 (−1.5, 6.0)	−2.5 *** (−3.6, −1.3)	−197.3 * (−350.3, −44.2)
Other race/multi-race, non-Hispanic	−91.6 (−189.7, 6.5)	−7.3 ** (−11.8, −2.8)	−2.3 * (−4.1, −0.5)	258.3 (−5.9, 522.5)
Hispanic	−90.8 * (−176.5, −5.1)	−5.6 * (−10.0, −1.1)	−2.7 *** (−4.1, −1.4)	−129.8 (−302.8, 43.2)
**Education**				
High school and below	Reference	Reference	Reference	Reference
College education and above	−11.7 (−77.7, 54.4)	−3.5 * (−6.8, −0.2)	0.00 (−1.1, 1.1)	1.4 (−134.0, 136.9)
**Marital status**				
Married	Reference	Reference	Reference	Reference
Divorced, separated, or widowed	2.7 (−69.8, 75.2)	−1.5 (−5.7, 2.7)	0.2 (−1.1, 1.5)	47.0 (−128.2, 222.2)
Never married	7.8 (−68.7, 84.2)	−0.3 (−3.9, 3.2)	−0.5 (−1.7, 0.8)	−6.2 (−167.8, 155.3)
**Income to poverty ratio (IPR)**				
IPR < 130%	Reference	Reference	Reference	Reference
130% ≤ IPR < 300%	−35.8 (−138.6, 67.0)	−1.0 (−6.9, 4.8)	0.1 (−1.4, 1.6)	−76.3 (−265.1, 112.4)
IPR ≥ 300%	−14.6 (−114.5, 85.4)	−4.0 (−9.2, 1.2)	0.2 (−1.3, 1.7)	16.5 (−177.7, 210.6)
**Obesity**				
Non-obese (BMI < 30)	Reference	Reference	Reference	Reference
Obese (BMI ≥ 30)	27.6 (−29.6, 84.9)	−1.7 (−4.8, 1.4)	0.8 (−0.2, 1.8)	128.6 (−3.1, 260.3)
**Smoking**				
Non-smoker	Reference	Reference	Reference	Reference
Former or current smoker	49.6 (−9.1, 108.3)	1.2 (−1.6, 4.1)	0.4 (−0.8, 1.5)	88.8 (−32.9, 210.5)
**Self-rated health**				
Good or excellent health	−25.0 (−106.7, 56.7)	3.0 (−0.2, 6.3)	−1.5 * (−2.9, −0.1)	−90.4 (−325.0, 144.2)
Fair or poor health	Reference	Reference	Reference	Reference
**Chronic condition**				
Diabetes	−46.1 (−140.2, 48.0)	−1.3 (−6.1, 3.6)	−0.2 (−1.9, 1.5)	−131.7 (−337.8, 74.3)
Arthritis	13.8 (−55.1, 82.8)	1.8 (−0.6, 4.2)	0.5 (−0.7, 1.7)	86.8 (−66.6, 240.1)
Coronary artery disease	−108.0 * (−213.0, −2.9)	2.3 (−5.1, 9.8)	−0.1 (−1.9, 1.8)	−211.6 (−477.0, 53.8)
Stroke	97.1 (−201.2, 395.3)	−3.5 (−9.4, 2.4)	0.9 (−3.6, 5.4)	391.8 (−242.5, 1026.1)
Cancer	−79.1 (−160.7, 2.5)	−1.6 (−6.8, 3.5)	−1.3 (−2.7, 0.1)	−193.9 * (−357.3, −30.6)
**Day of the week**				
Weekday	Reference	Reference	Reference	Reference
Weekend	88.6 ** (38.2, 139.0)	5.2 *** (3.2, 7.2)	0.8 (−0.2, 1.8)	51.1 (−62.5, 164.6)
**Survey wave**				
2005–2006	Reference	Reference	Reference	Reference
2007–2008	19.6 (−56.0, 95.2)	−2.7 (−6.6, 1.2)	0.6 (−0.7, 2.0)	62.7 (−81.5, 206.9)
2009–2010	−41.4 (−118.5, 35.7)	−1.4 (−5.7, 3.0)	−0.3 (−1.6, 1.0)	−53.8 (−197.4, 89.8)
2011–2012	1.3 (−81.5, 84.1)	0.7 (−3.6, 5.0)	−0.7 (−2.1, 0.7)	−68.8 (−237.2, 99.7)

Notes: Linear regressions were performed to estimate the adjusted differences in daily energy and nutrient intake at full-service restaurants by plain water and SSB consumption status, accounting for the NHANES multi-wave sampling design. * 0.01 ≤ *p* < 0.05; ** 0.001 ≤ *p* < 0.01; and *** *p* < 0.001.

## Abbreviations

The following abbreviations are used in this manuscript:

SSB	sugar-sweetened beverages
NHANES	National Health and Nutrition Examination Survey
NCHS	National Center for Health Statistics
FNDDS	Food and Nutrient Database for Dietary Studies
NCI	National Cancer Institute
CDC	Centers for Disease Control and Prevention (CDC)
FPED	Food Patterns Equivalents Database
IPR	income to poverty ratio
BMI	body mass index
CI	confidence interval
